# Necrotic Enteritis in Broiler Chickens: A Review on the Pathogen, Pathogenesis, and Prevention

**DOI:** 10.3390/microorganisms10101958

**Published:** 2022-09-30

**Authors:** Shahna Fathima, Walid Ghazi Al Hakeem, Revathi Shanmugasundaram, Ramesh K. Selvaraj

**Affiliations:** 1 Department of Poultry Science, The University of Georgia, Athens, GA 30602, USA; 2 Toxicology and Mycotoxin Research Unit, US National Poultry Research Center, Athens, GA 30605, USA

**Keywords:** necrotic enteritis, *C. perfringens*, broilers

## Abstract

*Clostridium perfringens* type A and C are the primary etiological agents associated with necrotic enteritis (NE) in poultry. The predisposing factors implicated in the incidence of NE changes the physical properties of the gut, immunological status of birds, and disrupt the gut microbial homeostasis, causing an over-proliferation of *C. perfringens*. The principal virulence factors contributing to the pathogenesis of NE are the α-toxin, β-toxin, and NetB toxin. The immune response to NE in poultry is mediated by the Th1 pathway or cytotoxic T-lymphocytes. *C. perfringens* type A and C are also pathogenic in humans, and hence are of public health significance. *C. perfringens* intoxications are the third most common bacterial foodborne disease after *Salmonella* and *Campylobacter*. The restrictions on the use of antibiotics led to an increased incidence of NE in poultry. Hence, it is essential to develop alternative strategies to keep the prevalence of NE under check. The control strategies rely principally on the positive modulation of host immune response, nutritional manipulation, and pathogen reduction. Current knowledge on the etiology, pathogenesis, predisposing factors, immune response, effect on the gut microbial homeostasis, and preventative strategies of NE in this post-antibiotic era is addressed in this review.

## 1. Introduction

Restrictions on the use of subtherapeutic doses of antimicrobials as antibiotic growth promoters (AGPs) in the broiler industry have led to the resurfacing of enteric diseases such as necrotic enteritis (NE). NE is described as a complex, multifactorial epizootiology that is presented as an acute clinical or subclinical disease [1]. The first report on NE in poultry was published in the early 1960s [2]. As per a survey conducted by Hughes et al. [3], due to the regulations on the use of antibiotics in the United Kingdom, the incidence of enteric and respiratory diseases was found to be significantly increased, and specifically the occurrence of NE, coccidiosis, wet litter, and respiratory diseases was considerably higher [3].

NE is caused by *Clostridium perfringens* and primarily affects chickens of 2 weeks to 6 months of age. The annual cost of NE is estimated to be USD 6 billion globally [4]. The enormous economic loss is associated with decreased production performance, increased mortality of up to 1% per day, disease treatment costs, and condemnation of carcasses in production plants due to cholangiohepatitis [5,6]. In humans, *C. perfringens* intoxications are the third most common bacterial foodborne disease after *Salmonella* and *Campylobacter*. The disease incidence in humans ranges between 359 and 2173 cases annually in the United States. Poultry and poultry products account for 30% of the outbreaks (43 outbreaks), and 92% of the outbreaks can be traced back to meat and poultry as a single identified food commodity [7].

In a study conducted in the UK among poultry farm managers, of the 75 respondents, 32.8% reported a case of NE in at least one flock. The study also demonstrated the strong association between wet litter, coccidiosis, and NE [8]. In Denmark, the prevalence of NE was reported in one to two of 1700 farms. However, this increased to 25 after the ban on antibiotics. The lower incidence of NE in Denmark can be attributed to the continued use of anticoccidial ionophores [9]. In a study conducted by Gaucher et al. [10], broiler chickens raised in a drug-free system had a daily weight gain 3.15% lower than those raised in the conventional system. Additionally, the incidence of NE was 0% in conventional flocks, whereas 27.4% of drug-free flocks experienced clinical NE, and 49% experienced subclinical NE [10]. Due to the economic impact of NE on the global broiler industry and production losses, it is essential to devise alternative strategies for preventing and controlling NE.

Recently, the research into antibiotic alternatives capable of improving the gut health and immune status of poultry has intensified. However, the alternatives currently available are not as effective as antibiotics in controlling NE. A greater understanding of the virulence factors of *C. perfringens*, pathogenesis of NE, and host responses are required to develop effective control strategies and a new generation of supplements. The virulence factors associated with NE, and pathogenesis of NE, are not fully understood, and these topics are being continuously investigated. This review focuses on the recent advancements in the understanding of the virulence factors of *C. perfringens*, the pathogenesis of necrotic enteritis, the immune response of the host, the interaction of the gut microbiota with *C. perfringens*, control strategies, and zoonotic risk.

## 2. Etiology

*C. perfringens* (types A and C), previously known as *C. welchii*, is the primary etiological agent linked with NE in poultry. *C. perfringens* is a boxcar-shaped, Gram-positive, encapsulated, anaerobic spore-forming bacteria that is a natural resident in the soil and the intestinal tract of warm-blooded mammals [11]. *C. perfringens* grows at temperatures ranging from 6 °C to 50 °C, with the optimum being between 43 °C and 47 °C. The optimum pH for the growth of *C. perfringens* is between pH 6.0 and 7.2. However, *C. perfringens* can also grow at a broad range of pH, varying from 5.0 to 9.0. *C. perfringens* spores are resistant to heat, desiccation, and radiation [12,13,14]. *C. perfringens* ferments glucose, lactose, fructose, maltose, mannose, galactose, sucrose, inositol, and starch, yielding acetic acid and butyric acid as products with or without butanol [15].

**Figure 1:**
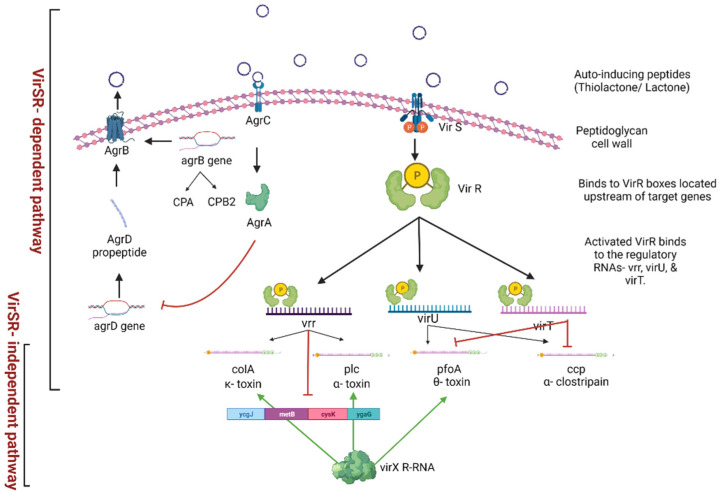
Regulation of virulence gene activation and toxin production by the quorum sensing dependent VirS–VirR regulatory system in *C. perfringens*. Created with Biorender.com (accessed on 30 May 2022).

*C. perfringens* infects live tissues, causing necrosis. *C. perfringens*, due to its rapid growth and metabolism, outcompetes other bacteria in the gut. The VirSR two-component regulatory system (Figure 1) in *C. perfringens* regulates the secretion of toxins such as hyaluronidases, sialidases, NetB, energy metabolism genes, catalytic enzymes, and transporters, causing the hallmark signs of necrotic enteritis [16]. Without a suitable nutrient substrate, *C. perfringens* forms dormant spores that can survive for long periods [11]. The ability to survive under varied environmental conditions and the vast array of virulence factors helps *C. perfringens* to establish itself successfully in the host.

## 3. Virulence Factors

The complete genome analysis of *C. perfringens* revealed the absence of genes related to amino acid biosynthesis, indicating that this bacterium cannot grow in an environment scarce in amino acids [11]. To overcome this challenge, *C. perfringens* possesses an arsenal of virulence factors that contributes to the degradation of host tissues and extraction of essential nutrients for *C. perfringens*’ survival within the host [17]. *C. perfringens* isolates vary in their production of these toxins, as no one isolate produces all of these toxins. This variation in toxin production created the toxinotype classification scheme that classifies *C. perfringens* into six groups (Table 1. *C. perfringens* acquires more than 20 toxins and enzymes (Table 2) that play a crucial role in its pathogenesis. These toxins are classified into four groups: (1) membrane-damaging enzymes, (2) pore-forming toxins, (3) intracellular toxins, and (4) hydrolytic enzymes [5,17].

### 3.1. Clostridium perfringens Alpha (CPA) Toxin

All *C. perfringens* isolates contain the alpha-toxin gene situated within the bacterial chromosomes. The alpha toxin is a zinc dependent-enzyme that has a phospholipase C activity. It consists of three domains, including the C-domain, which serves as the membrane-binding site; the N-domain, which contains the catalytic site; and the central loop domain, which uses the ganglioside GM1a binding site as a receptor [32].

The mode of action of alpha toxin is complicated, as it varies among cell types [33]. CPA toxin hydrolyses phospholipase C (PLC) and sphingomyelin (SM) in the eukaryotic cell membranes, yielding diacylglycerol (DAG) and ceramide, respectively. This leads to the activation of phospholipid metabolism, primarily the arachidonic acid pathway releasing thromboxane A2 (TXA2). CPA also interacts with the Gi-type GTP-binding proteins (Gi-GTP BPs), activating the endogenous host phospholipase (PI-PLC) and sphingomyelinase (SMase). The release of DAG, inositol triphosphate (IP3), ceramide, sphingosine, and sphingosine-1-phosphate(S1P) [32] by IP3 and S1P stimulates the release of calcium ions from the endoplasmic reticulum and Golgi apparatus, resulting in an increased intracellular calcium level. IP3 binds to the IP3 receptors in the presence of calcium ions, leading to the activation of the mitochondrial or intrinsic pathway of apoptosis [32,34]. CPA toxin interacts with the host cell surface receptor tropomyosin receptor kinase A (TrkA), which leads to the phosphorylation of PDK1 and PKCθ and subsequent activation of the MEK/ERK signaling cascade and NFκB, stimulating the production of reactive oxygen species and IL-8 [34]. Reactive oxygen species increase the oxidative stress in cells, resulting in cell death [35]. A high level of IL-8 increases neutrophil/heterophile attachment to the fibronectin, which slows their migration towards infected sites [36].

There are inconsistencies regarding the role of α-toxin in the pathogenesis of NE in chickens. An α-toxin null mutant was still virulent and capable of inducing necrotic enteritis in a chicken disease model [37]. Another study demonstrated that the α-toxin negative mutants could produce lesions of the same severity as the wild-type strain in chickens [37]. Additional research is required to completely understand the role of α-toxin in the pathogenesis of NE in chickens.

### 3.2. Clostridium perfringens Beta (CPB) Toxin

Beta toxin (CPB) is one of the most potent toxins produced by *C. perfringens* [19]. CPB is found on a large virulence plasmid found in *C. perfringens* type B and C isolates [19]. CPB is formed as a protoxin that is made up of 336 amino acids before a 27-amino acid signal sequence is removed to generate the secreted form of the toxin. CPB is a beta pore-forming toxin related to the alpha-hemolysin family [38]. CPB cell cytotoxicity is mediated through a pore formed on the membrane of the targeted cell [19]. The presence of a pore allows for an uncontrolled exchange of ions between the targeted cell and its environment, resulting in cellular homeostasis disruption that eventually leads to cell death [19].

### 3.3. C. perfringens Enterotoxin (CPE)

The *C. perfringens* enterotoxin gene can be carried on the plasmids or the chromosome. Nearly 5% of *C. perfringens* isolates have the CPE toxin, with type A being the most common [39]. CPE is a pore-forming toxin made up of 319 amino acids [23]. The CPE toxin contains two main domains, the C domain, which serves as a receptor binding domain, and the N domain, which mediates the oligomerization and pore formation [23].

The CPE gene is expressed exclusively during sporulation [40]. During the sporulation process, the CPE toxin accumulates inside the spore and is released once the mother cell is lysed. Upon the release of the CPE toxin from the spore, trypsin and chymotrypsin activate the CPE toxin by removing around 27–39 amino acids from its N-terminal sequences [40]. The CPE toxin shows a high affinity for tight junctions at the gut level, namely claudins [41]. The claudins family consists of 27 different proteins that play a role in maintaining the structure and function of tight junctions [42]. Claudins are formed as four transmembrane domains, with a long cytoplasmic tail (COOH-terminal) and two extracellular loops (extracellular loop one and extracellular loop two) [42]. Both extracellular loops play a crucial role in CPE binding to claudins. Claudins 3, 4, 6, 8 and 14 act as functional receptors for CPE to facilitate its binding to the host cell [41]. CPE interacts with receptor claudins and non-receptor claudins to form small complexes. These small complexes interact together to mediate the CPE oligomerization, which creates a pre-pore on the plasma membrane surface of the host cell known as CH-1 [41]. In the CH-1 complex, the beta-hairpin changes into a beta-barrel that forms the pore at the plasma membrane level [41]. Pore formation leads to a disruption in the plasma membrane permeability and increases calcium influx in the cells, which eventually leads to cell death [43]. CPE-mediated damage to the tissues aids the toxin in binding claudins and occludins to form a CH-2 complex. It is still unclear how CH-2 contributes to the cytotoxicity of the cells [41].

### 3.4. Necrotic Enteritis Like Toxin (NetB)

The necrotic enteritis B-like toxin is a novel toxin isolated from the *C. perfringens* strain causing NE in chickens [44]. The NetB toxin is found on a conjugative plasmid and belongs to the family of beta-pore-forming toxins [45]. The NetB toxin shares a similar heptameric assembly with the alpha-toxin produced by *Staphylococcus aureus* [45]. The NetB toxin’s interaction with the cholesterol situated in the membrane of the cells mediates the pore formation and oligomerization [45]. The pore formation disrupts the membrane and increases the influx of ions, which leads to cell death [45]. The molecular pathways of the NetB toxin in the intestine have not been fully explored. This hinders the development of NE mitigating tools.

Chickens challenged with a NetB mutant, or a mutant complemented with a NetB- plasmid, did not develop NE, whereas a wild-type strain and a NetB mutant complemented with NetB+ plasmid was able to produce the disease [44]. However, an assessment of the prevalence of NetB demonstrated that 41.7% of the 12 isolates recovered from chickens with NE were NetB-negative. The 12 *C. perfringens* isolates were obtained from four different flocks with NE. Furthermore, 24 isolates obtained from chickens without NE from four different farms were found to be NetB-positive [46]. Hence, it is still unclear and controversial as to which toxin is the cause of NE.

### 3.5. TpeL

The TpeL toxin was first identified in 2007 in a culture supernatant of *C. perfringens* type C [47]. The TpeL gene is located on a plasmid in *C. perfringens* type B, C, and sometimes A [47]. The TepL toxin belongs to a family of large clostridium toxins that are usually made of single-chain proteins, with an ABCD-domain form [48]. The A-domain is situated on the N-terminal of the chain, and it has glycosyltransferase activity; the B-domain is situated on the C-terminal of the chain and acts as the binding site; the C-domain is located after the A-domain and serves as the cysteine protease; the D-domain is the delivery domain that delivers the A-domain into the cell’s cytosol [48]. LDL receptor-related protein 1 (LRP1) mediates the endocytosis of TpeL, while the D-domain facilitates TpeL entry into the endosomal membrane [49]. Conformational changes in the toxin are induced by the acidic environment in the endosome, leading to channel formation [48]. The toxin breakdown occurs following the interaction between the C-domain (cysteine protease) and inositol phosphate. The A-domain is then released into the cytosol of the cell. The A-domain mediates the glycosylation of different GTPases that play a role in actin regulation/formation [48]. Blocking these essential proteins leads to the loss of cell morphology and intercellular connections, and eventually cell apoptosis [48].

## 4. Culture and Detection

**Figure 2:**
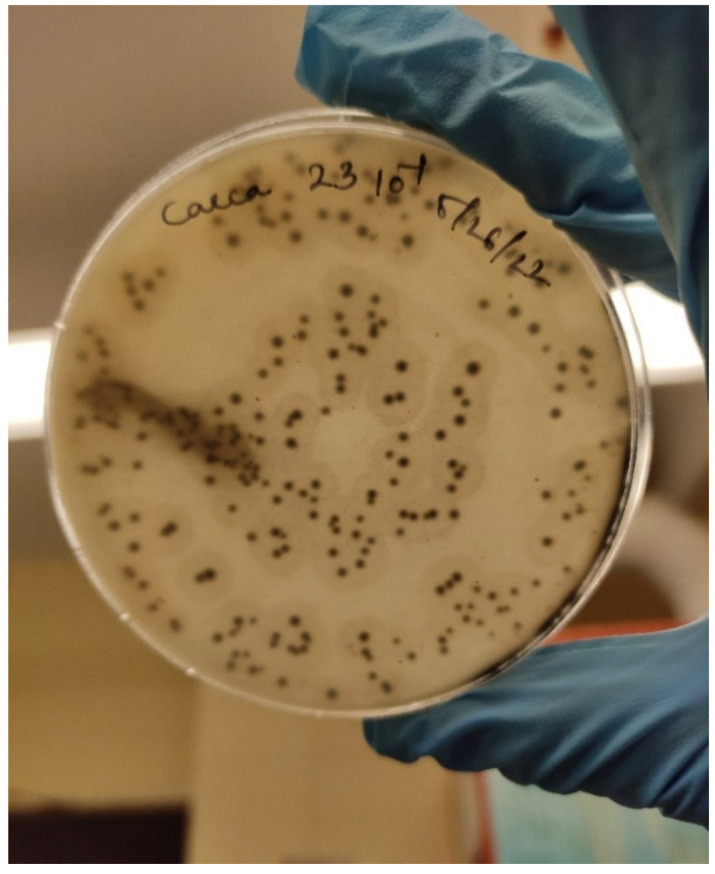
Growth of *C. perfringens* colonies on TSC agar supplemented with egg yolk emulsion.

The detection and quantification of *C. perfringens* can be performed by standard microbiological plating methods. The homogenized samples are serially diluted under aseptic conditions and are plated on selective media. The enrichment of *C. perfringens* in the sample can be performed by adding 1 mL of the homogenized sample to 10 mL of pre-reduced cooked meat medium and incubating under anerobic conditions for 24 h at 37 °C. *C. perfringens* selective agar (CPSA) supplemented with 0.5% yeast extract, 2.0% agar, 5% citrated bovine blood, 0.1% sodium metabisulfite, and 400 µg D-cycloserine/mL can be used for direct plating [50]. Minimal salt media consisting of solutions A and B, supplemented with alanine, glycine, methionine, or leucine (10 mg mL^−1^), have also been used for invitro culture of *C. perfringens*. The media must be pre-reduced and incubated under anaerobic conditions [51]. The pour plate method using perfringens tryptose-sulfite-cycloserine (TSC) and Shahidi–Ferguson perfringens (SFP) agar base mixed with egg yolk emulsion has also been described for the enumeration of *Clostridium perfringens* colonies [52]. Typical black colonies are produced by *C. perfringens* on TSC agar due to the reduction of sulfites. The halo around colonies in media supplemented with egg yolk is due to the lecithinase activity of *C. perfringens* (Figure 2). *C. perfringens* spore formation can be induced by inoculating the culture in Duncan Strong sporulation broth, boiling the broth at 100 °C, and then inoculating the boiled sample in reinforced Clostridial agar [53]. The presumptive positive colonies are confirmed by Gram staining, capsule staining, colony characteristics and biochemical tests, summarized in Table 3 [53,54]. This makes standard plate counts time-consuming and painstaking.

Conventional PCR and real-time PCR are more rapid and accurate methodologies with a wider range of detection. The end-point concentration of DNA in conventional PCR is 2–3 log10 and requires post-PCR processing, whereas real-time PCR has a much wider range of 5–6 log10 [55]. A study comparing the efficiency of culture-based methods and real-time PCR demonstrated that the PCR results correlated highly with the culture-based method and that the relative standard deviations (RSDs) of real-time PCR was lower, suggesting it to be a more accurate and reproducible approach [56].

## 5. Predisposing Factors

**Figure 3:**
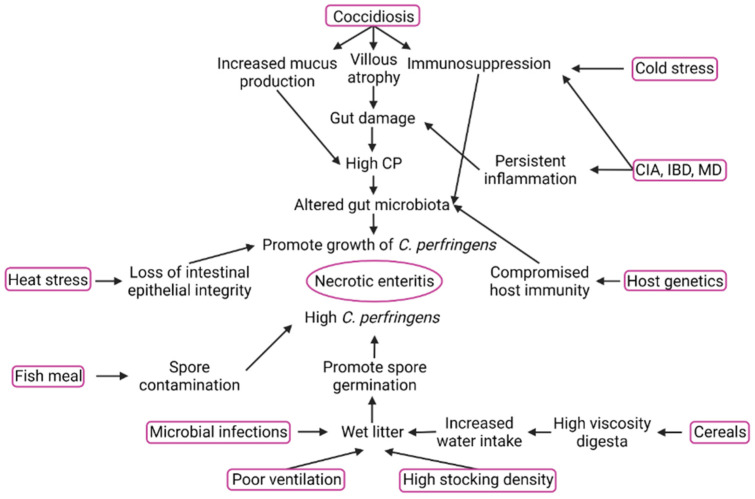
Predisposing factors of NE and their mechanism of action. Created with Biorender.com (accessed on 30 May 2022).

The presence of predisposing factors is necessary to produce a conducive environment for the proliferation and colonization of *C. perfringens* in chickens. These predisposing factors play a critical role in field disease outbreaks. The predisposing factors can (1) damage the intestinal epithelium, (2) increase mucus secretion, (3) disrupt gut microbiota composition, (4) change gut transit times, and (5) alter the immune status of host. These changes in the physical properties of the gut and the immunological status of birds cause an over-proliferation of *C. perfringens*, precipitating the disease [57,58]. For instance, challenging the birds with *C. perfringens* alone was incapable of producing NE, whereas fish meal-fed and *Eimeria*-challenged birds were infected significantly [59]. Some of the major predisposing factors contributing to the outbreak of NE under field conditions and employed in designing NE disease models for research are discussed here and are summarized in Figure 3.

### 5.1. Coccidiosis

Coccidia damages the intestinal epithelium, increases mucus production, and contributes to the rapid proliferation of *C. perfringens* by providing protein-rich nutrients derived from the damaged host tissue. *Eimeria* infection stimulates the host cell-mediated inflammatory response, resulting in a significant increase in intestinal epithelial mucogenesis, favoring the growth of mucolytic *C. perfringens*. *Eimeria* infection can also cause significant changes to the bird’s gut microbiome and immune status, further enhancing the chances of *C. perfringens* colonization [60].

In experimental models, care must be taken with the selection of species and dose of *Eimeria*, as *Eimeria* itself is highly pathogenic and causes mortality in birds. It is preferable to use species affecting the small intestine, such as *E. acervulina* or *E. maxima*, although *Eimeria* spp. affecting distal parts of the intestine are also demonstrated to predispose to NE [59,61,62]. Coccidia should be administered no earlier than 4–5 days before *C. perfringens* challenge to ensure the bacterial challenge coincides with the intestinal damage produced by Coccidia [63]. In a study conducted by Collier et al. [60], it was observed that the presence of *Eimeria* during *C. perfringens* infection significantly increased the necrotic intestinal lesions and mortality in chickens compared to the control. An increase in the abundance of anaerobic bacterial translocation to the liver and spleen was also detected [60]. These findings suggest that coccidiosis is an important factor predisposing the birds to NE. Hence, the prevention and control strategies for NE also include the control of coccidiosis.

### 5.2. Dietary Factors

Raw feed materials and their physical form considerably affect the pathogenesis of NE in chicken. In particular, feeds containing high levels of animal proteins and cereals rich in non-starch polysaccharides (NSPs) predispose the bird to NE by altering the viscosity of intestinal digesta and producing an environment suitable for the proliferation of *C. perfringens*. NSPs increase the water intake of birds, causing wet litter, and producing ideal conditions for the sporulation of *C. perfringens*, contaminating the litter [8]. In a study conducted by Riddell et al., the incidence of NE was more significant when the birds were fed hammermill-ground wheat compared to the group fed roller-mill-ground wheat, indicating the significance of the physical form of the raw material [64].

The source of protein in poultry feed significantly affects the pathogenesis of NE. Fish meal and potato protein concentrate, when used as a source of protein instead of conventional soybean meal, predisposes the birds to NE and results in a significantly higher lesion score [65]. Fish meal supplementation can alter the gut microbiota composition by (1) acting as a source of *C. perfringens*, (2) providing nutrients that promote *C. perfringens* growth, and (3) introducing biogenic amines, which cause proventriculus and gizzard erosion [59,66,67]. Dietary inclusion of increasing levels of fish meal result in a corresponding increase in the abundance of *C. perfringens* in the ileum of *C. perfringens*-challenged birds [56]. Hence, the protein source and concentration are highly important factors predisposing the birds to NE.

### 5.3. Feed Mycotoxins

Deoxynivalenol (DON), produced by the fungus Fusarium, is a common contaminant of poultry feed. Contamination of poultry feed by DON at a level of 3000 to 4000 μg/kg significantly increases the incidence of NE. DON contamination increases the total protein concentration of the duodenal content, favoring the growth and toxin production of *C. perfringens* [56]. Mycotoxins also decrease villus height, transepithelial electrical resistance, and increase the intestinal permeability of duodenum and jejunum, which is the principal site of mycotoxin absorption [68,69]. Similar effects were also observed with the mycotoxin fumonisin. Contamination of poultry feed with 20 mg/kg fumonisin exerts toxic effects on intestinal epithelial cells and induces microbial shift. Fumonisins reduce the length of the small intestine and decrease villus height and crypt depth [70]. The intestinal epithelium’s morphological and functional disruption predisposes the bird to NE.

### 5.4. Immunosuppression

Necrotic enteritis is most prevalent in chicks of about three weeks of age. This period corresponds to a decline in maternal antibodies which would otherwise protect the chicks from diseases [71]. Viral infections such as Marek’s disease, chicken infectious anemia, and infectious bursal disease predispose chicks to NE by altering the immune status of the bird [72]. Neonatal *Salmonella* Typhimurium infection also predisposes chicks to NE in laboratory challenge models. *Salmonella* infection possibly interferes with the host’s immunocompetence and causes persistent inflammation compromising intestinal integrity [73]. The use of commercial bursal disease vaccines or a combination of coccidia and bursal disease vaccines as a predisposing factor resulted in significantly higher lesion scores compared to *C. perfringens* challenge alone. Moreover, birds infected with NE had a considerably higher proportion of intrinsic *E. coli*, which might be attributed to the immunosuppression caused by the predisposing factors or *C. perfringens* infection [74]. In general, infections compromising the bird’s immunity, such as bacterial, viral, or parasitic diseases, can predispose the birds to NE.

### 5.5. Stocking Density

Stocking density is defined as the total live weight (kg) or the number of birds occupying a bird shed at the same time per square meter of usable area [75]. In simple terms, it can also be interpreted as the area occupied per bird. An increase in stocking density can decrease production costs by reducing labor, fuel, and equipment and increasing the number of birds per unit area. Hence, it is a central factor affecting profitability in poultry production. However, a high stocking density can raise animal health and welfare concerns. High stocking density will also affect the litter and air quality causing stress, lameness, growth retardation and decreased quality of poultry products. High stocking density is associated with a considerably higher number of *C. perfringens* in the caeca, altered gut microbiota, and significantly lower pH of intestinal digesta [76], all of which can contribute to the development of NE.

### 5.6. Temperature

Heat stress is defined as an increase in the environmental temperature and humidity above the comfort zone. The birds respond to heat stress by panting, spreading wings, and increasing the respiratory rate to dissipate heat [77]. Heat stress affects the health, performance, and welfare of poultry, their intestinal epithelial integrity, and alters their gut microbiota, predisposing the bird to enteric pathogen infections [78]. Experimentally, heat stress increases the incidence and severity of necrotic enteritis lesions. Heat stress triggers the outbreak of NE in unchallenged birds and increases the severity of NE in challenged birds [79], indicating the possible role of heat stress in triggering the occurrence of NE under field conditions.

Cold stress, similar to heat stress, can affect the health and welfare of poultry [80]. Cold stress (15 °C) significantly increases the number of NE-positive birds and the severity of NE lesions in challenged birds [81]. The mechanism by which cold stress predisposes the birds to NE is not fully understood. The exposure of chickens to low temperatures can induce immunosuppression [82], and can alter the gut microbiota, predisposing the birds to NE.

## 6. Pathogenesis

Pathogenesis describes the complex and dynamic host–pathogen interactions at a molecular level [83]. Understanding the pathogenesis is important to devise control measures scientifically. The pathogenesis of bacterial infections can be broadly categorized into six phases, which are: (1) the colonization of the site of predilection; (2) growth and proliferation; (3) the acquisition of nutrients; (4) the evasion of host defense mechanisms; (5) the injury to host tissues; and (6) transmission [84]. In the pathogenesis of a rapidly growing pathogen such as *C. perfringens*, these phases are dynamic, occur almost simultaneously, and are indistinct.

Intestinal epithelial cells secrete mucins which form a physical barrier to prevent the entry of pathogens. Colonization and degradation of the mucus layer are essential for *C. perfringens* to establish and develop necrotic enteritis. Oligosaccharides on the central glycosylated region of the mucin glycoproteins offer potential binding sites for bacterial adhesins. Pathogenic *C. perfringens* secrete bacteriocins such as perfrins capable of displacing commensal Clostridium species colonizing the intestinal mucosa [85]. *C. perfringens* possess glycoside hydrolases [86] capable of utilizing the oligosaccharide sugars as a source of energy and chitinases [87] capable of degrading mucins. The degraded mucus is possibly a source of nutrition for *C. perfringens* and enables *C. perfringens* to localize and form microcolonies on the mucosa [88]. Predisposing factors such as *Eimeria* stimulate a T-cell-mediated inflammatory response, resulting in increased mucogenesis by the host intestinal epithelial cells and release of essential amino acids by tissue damage. The released mucins and essential amino acids promote the growth and colonization of *C. perfringens* [60].

Once *C. perfringens* reaches a threshold density, the Agr-like quorum sensing system sends out small auto-inducing molecules that upregulate the expression of the VirS–VirR two-component regulatory system and virulence-related genes such as NELoc-1 involved in adhesion [16,89]. Following degradation of the mucus layer, pore-forming toxins secreted by *C. perfringens* gain access to the epithelial cells. *C. perfringens* secrete proteolytic and collagenolytic enzymes that damage the basal domain of the epithelia, progressing towards the intercellular junctions, and spreading across the lamina propria. Consequently, the intestinal epithelium becomes necrotic and sloughs off [90]. The Agr-like quorum sensing regulated secretion of alpha toxin and perfringolysin aids in *C. perfringens* biofilm formation on the exposed submucosa [91]. Biofilms are bacterial populations surrounded by a matrix, forming a cohesive three-dimensional polymer network that facilitates bacterial adhesion to surfaces, provides mechanical stability, and protects the bacteria from host immune responses and antibiotics [91,92].

An intact intestinal epithelium is essential for absorbing and utilizing nutrients and preventing the entry of pathogens. The adjacent cells of the intestinal epithelium are held together by tight junctions, which maintain the intestinal integrity by sealing the paracellular space [93]. The tight junction proteins, particularly claudin-3 and claudin-4, act as functional receptors for *C. perfringens* enterotoxin (CPE) [94]. CPE binds to the claudin proteins, forming a ‘small complex’ approximately 90 kDa in size, which is insufficient to cause cytotoxicity [95,96]. Further, several small complexes cluster together, and their interactions lead to CPE oligomerization and pre-pore formation on the cell membrane [97]. An oligomer consisting of the CPE hexamer, several receptors, and non-receptor claudins form a ‘large complex’ called CH1 with a size of 450 kDa [95]. The β-hairpin loops of CPE assemble on the cell membrane to form a β-barrel, which inserts into the cell membrane, forming a cation permeating pore [98]. The pore formed by CPE permits calcium influx, causing cell death [98,99].

The gross lesions of necrotic enteritis are typically restricted to the jejunum and ileum but can also occur in the duodenum and ceca. The small intestine is usually thin, friable, and distended with gas. The tan-orange pseudomembrane provides the intestine’s typical “dirty Turkish towel appearance” in necrotic enteritis [100,101]. Pseudomembrane or diphtheritic membrane formation is frequently observed in the jejunum and less often in the duodenum, ileum, and ceca [101]. Subclinical necrotic enteritis is associated with hepatitis or cholangiohepatitis [102] and the gall bladder being distended with a flocculent substance [103]. Bile acids promote sporulation and enterotoxin production by *C. perfringens* [104], justifying the higher incidence of necrotic lesions in the upper parts of the small intestine, the duodenum, and jejunum.

## 7. Immune Response to Necrotic Enteritis in Broilers

### 7.1. Gut-Associated Lymphoid Tissue and Immune Response in Chicken

The avian immune system can broadly be classified into the innate and adaptive immune systems. The components of the innate immune system are monocytes, macrophages, dendritic cells, neutrophils, and natural killer cells and molecules such as antimicrobial peptides, nitric oxide, cytokines, and chemokines secreted by these cells. The adaptive immune system comprises B-lymphocytes and T-lymphocytes [105]. The avian lymphoid organs include central immune organs, namely the thymus, bursa of Fabricius, and bone marrow, and the peripheral immune organs are the spleen and cecal tonsils. In addition, there are single or multiple lymphoid follicles dispersed in the lamina propria of the intestine and bronchi, and these are termed gut-associated lymphoid tissue (GALT) and bronchial-associated lymphoid tissue (BALT), respectively [106].

The gut mucosa and GALT are constantly exposed to antigens of food, gut microbial, and pathogenic bacterial origin. Hence, the principal function of GALT is to prevent the entry and dissemination of pathogens. Non-specific barriers such as peristalsis, lysozyme, antimicrobial peptides, bile salts, gastric secretions, pancreatic secretions, and commensal gut microbiota are the primary defense mechanisms that prevent the entry of pathogens into the gut and pathogen’s systemic dissemination [107]. The GALT is composed of organized lymphoid tissues consisting of Meckel’s diverticulum, cecal tonsils, payers patches, bursa of Fabricius, regional lymphatics, and mesenteric lymph nodes, and dispersed lymphoid aggregates across the gut lamina propria and intraepithelial leukocytes [108]. The components of GALT can broadly be classified into mucosal inductive sites that encounter the environmental antigens and mucosal effector sites such as the lamina propria [109]. The outermost layer of GALT consists of the mucus layer, enterocytes, and intraepithelial lymphocytes separated by a basement membrane from the underlying lamina propria. The lamina propria comprises the submucosa and lymphocytes such as mast cells, dendritic cells, macrophages, natural killer cells, fibroblasts, and the draining lymphatics and lymph nodes [109,110,111]. The Payer’s patches are a cluster of B cells creating a follicle divided by T cell-rich segments and randomly dispersed in the wall of the small intestine. The intestinal epithelium overlying Payer’s patches, called the follicle-associated epithelium, consists of specialized cells named microfold cells (M cells), which actively endocytose and pinocytose the gut luminal antigens. The Payer’s patches form a ‘dome’-like structure, and the epithelium is relatively devoid of mucus, enabling effective sampling of the gut luminal antigens and their delivery to the underlying lymphoid tissue [112,113]. The intestinal epithelial cells, intraepithelial lymphocytes, and lamina propria lymphocytes secrete and respond to various cytokines, including TGF-α, TGF-β, IFN-γ, IL-1, IL-2 [114], IL-3, IL-4, IL-5, IL-6 [115], IL-8, IL-15 [116], and GM-CSF [114].

### 7.2. Innate Immunity

The gut’s mucosal immune system plays a significant role in the innate immune response to necrotic enteritis. Pathogen recognition receptors (PPRs) present on intestinal epithelial cells and cells of the innate immune system recognize conserved molecular structures on the pathogen, known as pathogen-associated molecular patterns (PAMPs) [117,118]. Toll-like receptors are one of most prominent PRRs expressed on cell surfaces, endocytic vesicular membranes, and other intracellular organelles. PRRs recognize the molecular motifs associated with the pathogen, such as flagellin, lipopolysaccharides, peptidoglycans, and bacterial genetic materials, and initiate innate immune responses to the pathogen. The chicken TLR repertoire comprise 10 genes, of which three are unique to chickens [119]. The expression of TLRs varies depending on the tissue and cell type, as summarized in Table 4, and this influences the type of immune response elicited in different tissues [120]. Other important PRRs are the C-type lectin-like molecules, cytosolic receptors, and RIG-I-like receptors [121].

At the gut mucosal interface, the enterocytes, intraepithelial lymphocytes, and innate immune cells express PRRs which are activated on interaction with the PAMPs, initiating a series of downstream signaling pathways which induce the production of reactive oxygen species, reactive nitrogen intermediates, and proinflammatory cytokines. Activation of the antigen-presenting cells upregulates the expression of co-stimulatory molecules, which are essential for developing adaptive immune responses [127,128]. TLR activation results in the recruitment of adaptor proteins to the intracellular domain of the TLR, further recruiting the interleukin-1 receptor-associated kinase (IRAK). This activates the tumor necrosis factor receptor-associated factor 6 (TRAF6), an essential ubiquitin E3 ligase. TRAF6 subsequently activates transforming growth factor-β-activating kinase (TAK1). TAK1 induces the phosphorylation of IκB or activates the kinases MKK3 and MKK6 and leads to the activation of the transcription factors NFκB and Janus kinase (JNK), respectively. The transcription factors NFκB and JAK translocate to the nucleus and induce the expression of genes necessary for the immune response [129]. The activation of TLRs on the APCs results in the upregulation of co-stimulators, MHCs and gene expression, inducing pro-inflammatory cytokines and chemokines. These activated APCs migrate to the regional lymphoid organs and facilitate the activation of naïve T-cells [130]. TLR activation also induces the production of antimicrobial peptides such as β-defensins and cathelicidins, which are the effector molecules of the innate immune system [131].

In chickens, the cell wall peptidoglycans of Gram-negative bacteria are recognized by TLR2b, TLR4 recognizes the lipopolysaccharide component of Gram-positive bacterial cell membrane, and TLR5 recognizes flagellin of both Gram-positive and Gram-negative bacteria [132]. *C. perfringens* challenge increases the expression of TLR1.2, TLR2.1, TLR4, TLR7 and TLR 15 in the spleen and ileum [133]. Likewise, NE upregulates the TLR2 and TLR15 mRNA expression in the duodenum. In vitro, *C. perfringens* peptidoglycan significantly increases the mRNA expression of IL-6, IL-8, TNF-α, and iNOS [134]. These results suggest the importance of TLRs in the innate immune response to *C. perfringens* challenge.

### 7.3. Adaptive Immunity

Activation of the APCs such as dendritic cells, macrophages, and B cells by the binding of ligands to PRRs upregulates the expression of MHC class II molecules and co-stimulators on the APCs. The activated APCs subsequently activate the naive T cells and promote their differentiation to different subsets of T-helper cells [135]. Activated macrophages secrete IL-12, stimulating the production of IFN-γ, which subsequently induces the differentiation of activated T cells to the Th1 subset, initiating the cell-mediated immune response [136]. IL-2 secreted by the activated T-cells stimulates effector T cells and regulatory T cells [137]. A study reported increased mRNA expression of IL-4, IL-10 and IFN-γ in birds challenged with *C. perfringens* [60]. The cytokine IFN-γ is associated with macrophage activation, differentiation of naïve helper T-cells to Th1 subset, and expression of MHC class II [138], whereas IL-4 and IL-10 are immunomodulatory cytokines that suppress the production of proinflammatory cytokines such as IL-12 and IFN-γ [139].

Interleukin 17A, produced by the Th17 cells, plays a significant role in antimicrobial defense at mucosal barriers by stimulating neutrophil recruitment, the secretion of antimicrobial peptides, and IgA production [140,141]. To compensate for the tissue injury due to infection and inflammation, the cytokines IL-17 and IL-22 promote the regeneration of intestinal epithelial cells. Subclinical NE activates Th2- and Th17-mediated immune responses, characterized by the upregulation of IL-1β, IL-13, and IL-17 cytokine expression in the cecal tonsils and jejunum.

On the other hand, some data suggest that the NE infection is resolved by a Th1 response mediated by the cytotoxic T-cells. The T-cell response to NE infection peaks on day 16, characterized by an increase in the numbers of T-helper cells, cytotoxic T cells, and double positive T cells [142]. In a study conducted by Park et al. [143], it was demonstrated that infection with *C. perfringens* induced the expression of IL-1β, IL-2, IL-12, IL-13, IL-17, IFN-α, IFN-γ, and TGF-β. However, it is noteworthy that the coinfection of *Eimeria* maxima and *C. perfringens* reduced the expression of the above-mentioned immune mediators significantly. Expression of iNOS was also significantly reduced in coinfected birds. This can be explained by the fact that the expression of IFN-γ, which mediates the expression of iNOS in epithelial cells and macrophages [144], is suppressed under coinfection with *Eimeria* and *C. perfringens* [143].

In general, the host immune responses and parameters vary widely between studies on necrotic enteritis models. The variation may be attributed to the different strains of *C. perfringens* used, modifications of the necrotic enteritis models, and the predisposing factors. The reactivity of pooled immune serum derived from broiler chickens immune to virulent *C. perfringens* to CP4, CP5, and CP6-derived secreted proteins was significantly different [145]. Additionally, the predisposing factors used in the disease models, such as pre-infection with *Eimeria* spp. and inclusion of fish meal in the diet, influence the severity of necrotic enteritis and the immune response elicited [146]. Other factors such as the timing of challenge, the use of a coccidial vaccine or virulent *Eimeria* spp., the dose of challenge, and research intention [147] will affect the immune response.

## 8. Microbial Shift during Necrotic Enteritis

The onset of necrotic enteritis is associated with a shift in the gut microbiota [148]. The various predisposing factors implicated in triggering necrotic enteritis directly or indirectly alter the gastrointestinal microbiome of poultry. Hence, it is important to understand the interaction between gut microbiota and pathogenic *Clostridium perfringens* in the development of necrotic enteritis.

In the group challenged with *C. perfringens* without predisposing factors, an increase in the abundance of *Candidatus savagella* was observed. *C. savagella* is a segmented bacteria found in the gastrointestinal tract of vertebrates, including chickens, and plays a significant role in immunomodulation [59]. *C. savagella* is reported to increase the number of epithelial lymphocytes, particularly Th17 cells, to promote their activation and maturation. *C. savagella* also induces the secretory IgA responses. The segmented filamentous bacteria were observed to establish a close interaction between the luminal side of Payer’s patches and ileal mucosa. This might explain the role of segmented filamentous bacteria in developing adaptive immune responses associated with the gastrointestinal immune system [149]. Only the group challenged with *C. perfringens* in the absence of predisposing exhibited an increase in the abundance of *C. savagella*, a suppressed *C. perfringens* infection, and did not exhibit any clinical symptoms of NE [59]. This observation emphasizes the role of *C. Savagella* in immune responses of poultry, and in field conditions, it is potentially capable of protecting birds from NE in the absence of other stress factors.

The group challenged with *C. perfringens* in the presence of fish meal revealed no change in the abundance of total *Lactobacillus* counts. However, a significant shift in the species composition of *Lactobacillus* was observed with an increase in the abundance of *L. reuteri* and *L. animalis* and a reduction in the abundance of *L. acidophilus* and *L. johnsonii* [59,61]. *L. acidophilus* is widely used as a probiotic capable of stimulating the immune system, pathogen antagonism, production of antimicrobials, and beneficially alter the metabolism of gut microbiota [150]. Therefore, an alteration in the gut microbiota and particularly a reduction in the abundance of beneficial bacteria may predispose the birds to NE. Although *L. reuteri* is generally recognized as a probiotic species, *L. reuteri* produces the antimicrobial peptide reuterin [151] which might eliminate the sensitive gut microbiota and alter the microbial population predisposing the birds to necrotic enteritis [152]. In the fish meal-fed group, the gut pH shifted from slightly acidic to neutral, which might be due to the high-protein diet. A high-protein diet causes a significant increase in isobutyrate, isovalerate, propionate, formic acid, and ammonia and a reduction in acetate and butyrate [61].

No significant shift in the microbiota was observed in the group challenged with *Eimeria*. However, a reduction in the gut microbial diversity was reported. The family most affected was *Ruminococcaceae* [61]. This is in agreement with the results of a study conducted by Li et al. [153], in which the ileal abundance of *Ruminococcaceae* and *Lachnospiraceae* was significantly reduced in birds challenged with *C. perfringens*. Members of the family *Ruminococcaceae* and *Lachnospiraceae* are the major butyrate producers of the chicken hindgut [154]. Butyrate has anti-inflammatory action and is reported to strengthen intestinal integrity by regulating the expression of tight junction proteins and epithelial cell proliferation [155]. This anti-inflammatory effect is achieved by activating the enteroendocrine cells and stimulating the secretion of glucagon-like peptide, which promotes intestinal blood flow, digestion, and the absorption of nutrients, and facilitates tissue repair [156].

**Figure 4:**
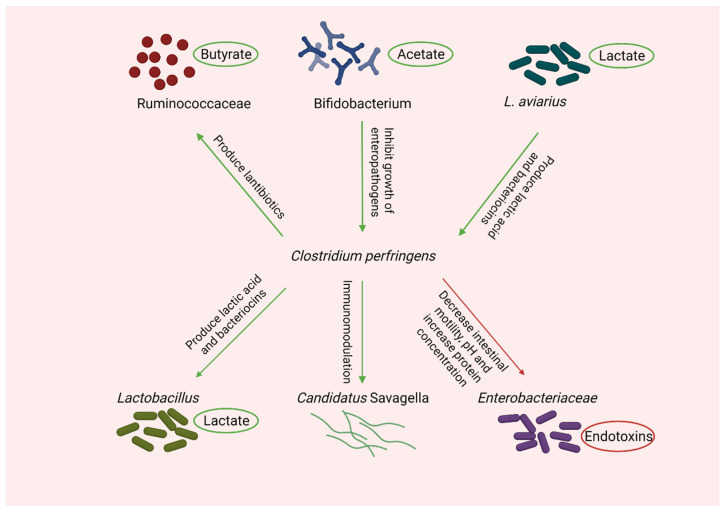
Gut microbial shift during NE challenge in chickens. Created with Biorender.com (accessed on 31 May 2022).

A different study reported a significant increase in the abundance of *Clostridium*, *Enterococcus*, *Streptococcus*, and *Turicibacter* in the presence of *Eimeria* [157]. There were no significant changes in pH and SCFA in the *Eimeria* group. However, a reduction in the proportion of formic acid and propionic acid was observed [61]. A general shift in the chicken intestinal microbiota associated with necrotic enteritis is summarized in Figure 4. In short, the shift in microbiota, pH, and SCFA largely depends on the predisposing factors used in experimental chicken models.

## 9. Zoonosis

Poultry is the largest source of animal protein the United States, valued at USD 46.3 billion, of which broiler production accounts for USD 31.7 billion. The United States Food and Drug Administration encourages avoiding the use of antibiotics in food animals to combat antimicrobial resistance [158]. There has been an increase in the incidence of enteric diseases in poultry, such as necrotic enteritis in farms that stopped using antibiotics [159]. However, there is an increased public preference for ‘no antibiotic ever chicken’. In a study conducted by Craven et al. [160], it was observed that of the 16 broiler flocks from four different farms, 15 (94%) were found to be positive for *C. perfringens*. The sources of contamination varied from the paper pads from the hatchery used for the transport of chicks to the worker’s boots [160]. Hence, it is of paramount importance to reduce the incidence of enteric pathogens, particularly zoonotic pathogens.

Necrotic enteritis in poultry is caused by alpha-toxin-producing *C. perfringens* type A and, to a lesser extent, alpha and beta-toxin-producing *C. perfringens* type C [11]. In humans, *C. perfringens* type A and C are pathogenic. Some strains of *C. perfringens* type A produce CPE during sporulation, which is responsible for human foodborne infection [161]. The recovery rate of *C. perfringens* from chilled carcasses is over 81% [160], and it can potentially cause a public health hazard unless appropriate control measures are in place. Most cases of *C. perfringens* outbreaks in humans are associated with the incorrect temperature of cooked food. *C. perfringens* spores can persist in cooked food and germinate during the cooling down and storage of prepared foods [162]. Due to the rapid proliferation of *C. perfringens* (doubling time less than 10 min) and the wide temperature range at which they can grow, *C. perfringens* can reach levels that cause food poisoning faster than any other foodborne pathogen [163].

The use of antimicrobials as antibiotic growth promoters to improve the health and production in the poultry industry has led to the development of antimicrobial resistance among organisms such as *C. perfringens*, which are normal inhabitants of the poultry gastrointestinal tract [164,165]. In a study by Silva et al., 47.3% and 41.8% of *C. perfringens* isolates from broiler chicken intestines were found to be resistant to bacitracin and tetracycline, respectively [166]. A similar study reported phenotypic resistance of *C. perfringens* to gentamicin among 44% of the isolates, followed by bacitracin (40%) and tetracycline (40%). Additionally, most of the isolates were found to be multidrug-resistant. Genetic profiling of the isolates revealed the prevalence of the tetracycline resistance gene in 41.33% of the isolates, followed by 34.66% for erythromycin and 17.33% for bacitracin resistance genes [167]. These studies emphasize the necessity for reducing the incidence of foodborne pathogens and finding efficient alternatives to antibiotics.

## 10. Antibiotics Resistance in *C. perfringens*

*C. perfringens* is a normal inhabitant of the gastrointestinal tract of animals. With the extensive use of antibiotics in poultry production, *C. perfringens* emerged as a reservoir of antibiotic resistance genes. These resistance plasmids are transferrable to other pathogenic bacteria in the gut by conjugation [168]. Antibiotics such as tylosin, bacitracin, lincomycin, penicillin, and tetracycline are commonly used in poultry and swine production for the prophylactic and therapeutic management of *C. perfringens*-induced neonatal diarrhea and NE, respectively. The first conjugative plasmid identified to confer antibiotic resistance in *C. perfringens* was pIP401, which imparts resistance to chloramphenicol and tetracycline. The most common is the tetracycline resistance plasmid, which is associated with the use of antibiotics in livestock feed [169]. The data from studies suggest that the tetracycline resistance plasmids isolated from different host species have a common pCW3-like progenitor, which is also isolated from humans [17]. Resistance to chloramphenicol is encoded by the *catP* gene and is mediated by the production of enzyme chloramphenicol acetyltransferase which prevents binding of chloramphenicol to the bacterial ribosomes. Bacitracin-resistant *C. perfringens* were isolated from avian NE, and the resistance determinant was located on the pJIR4150 conjugative plasmid. The resistance was mediated by the active efflux of bacitracin from the cell [170]. Lincomycin resistance in *C. perfringens* results from the activation or inactivation of the enzyme lincosamide nucleotidyltransferase encoded by the *lnu* gene family [168].

*C. perfringens* are susceptible to penicillin, metronidazole, meropenem, cefoxitin, piperacillin-tazobactam [171], ceftiofur, avilamycin, virginiamycin, and nosiheptide. Nevertheless, the prevalence of resistance genes varies depending on the geographical region as well. Of the 269 *C. perfringens* isolates, 9.5% were found to be resistant to metronidazole in Thailand [172]. Oxazolidinones are a novel class of synthetic antimicrobial agents and is used in the treatment of Gram-positive bacterial infections, particularly MRSA, VRE, and penicillin-resistant pneumococci [173]. The first report of the mobile oxazolidinone/phenicol resistance gene *optrA* in chicken *C. perfringens* isolates was in China. Recent studies also reported an association between the presence of toxin encoding genes such as *netB*, *cpb2*, and *tpeL* and antimicrobial resistance in *C. perfringens*. The use of antibiotics is thought to select for virulence and antibiotic resistance simultaneously [174].

## 11. Control of Necrotic Enteritis in the Post-Antibiotic Era

Antibiotics at subtherapeutic doses were used in poultry feed to enhance the health and performance of broiler chickens. Antibiotics modify the gut microbial composition, decrease bacterial catabolism and fermentation, reduce intestinal wall thickness [175], improve nutrient availability, and enhance the bird’s health, thus decreasing the incidence of enteric diseases [176]. Restrictions on the use of antibiotics have led to the re-emergence of effectively controlled poultry enteric diseases that cause massive production losses [177]. A thorough understanding of the pathogenesis and interaction between nutrients, gut microbiota, and NE is essential to prevent and reduce the incidence of NE in poultry. Several alternatives to in-feed antibiotics such as such as probiotics, prebiotics, dietary modifications, short chain fatty acids, plant extracts, and essential oils have been employed to control the incidence of NE. These approaches rely on the positive modulation of host immune response, nutritional manipulation, and pathogen reduction [178]. Additionally, biosecurity measures and vaccination strategies should be established on farms to limit the exposure of birds to pathogenic organisms.

### 11.1. Probiotics

Probiotics are defined as “live microbial feed supplements which, when administered in adequate amounts, beneficially affect the host animal by improving the gut microbial balance” [179]. The proposed modes of action of probiotics are: (1) competitive exclusion and antagonism, (2) improving the host intestinal health and integrity, (3) improving digestion and absorption, (4) enhancing growth and performance, and (5) immunomodulation [180]. An ideal probiotic should be non-pathogenic, of host origin, free of adverse side effects, should have demonstrated a beneficial effect on the host, be resistant to gastric acid and bile salts, be able to adhere to the host gastrointestinal mucosa, competitively exclude pathogenic bacterial colonization, be sensitive to antibiotics, positively modulate host immune functions, and be able to withstand processing and storage conditions [181,182,183]. Probiotics can be composed of a single strain or a combination of different strains of beneficial bacteria [184].

Probiotics supplementation reduces the colonization of enteric pathogens in poultry [185]. In a study conducted by Shanmugasundaram et al., it was reported that probiotic supplementation significantly increased the anti-*C. perfringens*-specific IgA and reduced the cecal load of *C. perfringens* in chickens [186]. In another study, supplementation of *Butyricicoccus pullicaecorum* during NE challenge significantly reduced the lesion score and improved growth performance in birds challenged with *C. perfringens* [187]. Probiotic supplementation reduces the oxidative damage, and intestinal cell apoptosis associated with NE challenge. Supplementation of *L. johnsonii* is associated with enhanced intestinal immunity in the NE challenge model [188]. The abovementioned beneficial effects of probiotic supplementation in ameliorating the damage caused by NE makes probiotics a potential alternative to antibiotics in the control of NE and other economically critical enteric diseases in poultry.

### 11.2. Prebiotics

Prebiotics are generally defined as “selectively fermented feed ingredients that cause specific changes in the composition and activity of the gastrointestinal microbiota, thus conferring benefits upon host health” [189]. For a feed ingredient to be considered as prebiotic, it should be resistant to hydrolysis by mammalian enzymes and gastric acids and absorption in the upper gastrointestinal tract, be fermentable by intestinal microbiota, provide selective stimulation of intestinal microbiota beneficial for the health and wellbeing of the host, and provide positive modulation of the gut mucosal immune response against pathogen invasion [183,190]. The stimulation of the gut mucosal immune system [191], the inhibition of pathogen colonization, and the production of SCFAs [192] are the mechanisms by which prebiotics exert their beneficial effects in the host gut health and integrity. Some of the most frequently used prebiotics in the poultry industry are mannooligosaccharides (MOS) [193], fructo-oligosaccharides (FOS) [176], inulin [194], and arabinoxylans [195].

The yeast cell wall is a good source of MOS, D-mannose, β-glucans, and α-methyl-D-mannoside [196]. Supplementation with yeast cell wall extract favors the growth of beneficial gut microbiota and regulates the stimulation of proinflammatory pathway in chickens [197]. In a study conducted by our lab, supplementation of killed whole yeast cells was found to increase the percentage of Tregs and the expression of IL-10 mRNA and decrease the expression of IL-1 mRNA in the cecal tonsils of broilers [198]. Supplementing FOS and MOS in the diet is also reported to increase the diversity of beneficial gut microbiota and decrease the abundance of *E. coli* and *C. perfringens* in the ileum of broilers [176]. Another study has demonstrated that supplementing probiotics (30%) and prebiotics (29%) reduced mortality due to NE to a level comparable to that of in-feed antibiotics (39%) [199]. These results, and the fact that additives such as prebiotics and probiotics are generally devoid of adverse side effects, make them a better alternative to antibiotics in the controlling of enteric diseases in poultry.

### 11.3. Phytobiotics

Phytobiotics or phytogenics are plant-derived bioactive compounds used in poultry feed owing to their therapeutic value. Phytobiotics can be broadly classified as (1) Botanicals, which are processed whole or part of plants, (2) essential oils, which are volatile plant extracts obtained by hydro distillation [200], (3) herbs, which are flowering, non-woody, non-persistent plants [201], or (4) oleoresins, which are plant extracts based on non-aqueous solvents such as carvacrol, capsicum, and cinnamaldehyde [202]. Plant extracts are secondary plant metabolites that typically belong to classes of glucosinolates, isoprene derivatives, and flavonoids [203]. These compounds exhibit antimicrobial [204], antioxidant [205], anti-inflammatory [206], immune modulation [207], and anti-parasitic properties [205,208]. Supplementation of plant extracts in chicken diets stimulates the endocrine system [209], improves small intestinal digestion, and maintains gut microbial homeostasis [210].

Phytobiotic supplementation is reported to increase villus height, villus–crypt ratio, jejunal surface area [211], and the abundance of lactic acid-producing bacteria in broilers [212]. Supplementation of *Allium hookeri* leaf and root extracts increases splenic lymphocyte proliferation, nitric oxide production by splenic macrophages, and suppresses tumor cell proliferation in chickens [213]. Furthermore, phytobiotic supplementation ameliorates intestinal lesions, oocyst shedding, loss of body weight, and downregulates the secretion of pro-inflammatory cytokines in the NE challenge model [214]. As a natural additive, phytobiotics can be a great replacement for antibiotics. However, a clear understanding of their mechanism of action, toxicity, interactions with other feed ingredients, and safety assessment requires completion before their extensive use in the poultry feed industry.

### 11.4. Organic Acids

Organic acids are traditionally used as preservatives and decontaminants due to their antibacterial properties [215]. Short chain fatty acids such as acetic acid, propionic acid, butyric acids and formic acids, medium chain fatty acids (MCFAs) such as capric acid, caprylic acid, caproic acid, and lauric acid [216], and carboxylic acids such as lactic acid, citric acid, fumaric acid, tartaric acid, sorbic acid, and malic acid are the most commonly used dietary organic acids in the poultry industry [217]. The mode of action of dietary organic acids in poultry are not completely elucidated; however, the proposed mechanisms are: (1) decreasing the pH of the GI tract, especially the upper GI tract, consisting of the crop, proventriculus and gizzard [218]; (2) improving nutrient utilization by activating zymogens and decreasing gastric emptying [219]; and (3) inhibiting the growth of pathogens by penetrating bacterial cell walls and disrupting normal cellular functions [220].

Individual organic acids [219] or a blend of different organic acids [216] are used as an alternative to antibiotic growth promoters in poultry feed. Supplementation of a blend of SCFAs and MCFAs during NE challenge improves the serum IgA concentration, feed efficiency, apparent ileal digestibility of nutrients and footpad health of chickens [221]. Likewise, dietary organic acid supplementation improves FCR, attenuates mucosal atrophy and enterocyte necrosis associated with NE, and upregulates the expression of jejunal tight junction proteins. Thus, dietary organic acid supplementation promotes gut health and positively modulates the gut microbiota, inhibiting the proliferation of pathogenic bacteria and the occurrence of enteric diseases in broiler chickens [200,221]. Hence, dietary organic acids are a prospective candidate for preventing NE in broilers.

### 11.5. Immunoglobulins

Chicken is used as an immunization host for the production of antibodies. The IgY antibody, otherwise known as ‘immunoglobulin of egg yolk,’ which is equivalent to the mammalian IgG, is the primary chicken serum immunoglobulin. The concept of egg yolk immunoglobulins exploits the process of the transfer of maternal antibodies from the serum to the egg yolk in high concentrations [222]. Pure maternal antibodies or egg yolks from immunized chickens containing pathogen-specific maternal antibodies can be used as a feed additive to protect chicks [223]. These pathogen-specific egg yolk antibodies can neutralize enteric pathogens and hence decrease the incidence or ameliorate the pathologic effects of enteric diseases in poultry [224].

Chicken egg yolk-derived antibodies and hyperimmunized egg yolk powder reduce the colonization by enteric pathogens such as *E. coli* O157:H7 [225], *Salmonella* Enteritidis, *Salmonella* Typhimurium [226], *Campylobacter jejuni* [227], *C. perfringens*, and infectious bursal disease virus [228]. Oral administration of anti-clostridial IgY increases feed intake and improves weight gain in infected birds. It also decreases morbidity and mortality in infected birds [224]. Hence, anti-clostridial IgY harvested from egg yolk is used as an alternative to antibiotics for the passive immunization of broiler chickens against NE outbreak. However, approval from the regulatory authorities is required for the commercialization of hyperimmunized egg yolk powder in the poultry industry.

### 11.6. Bacteriophages

Bacteriophages are viruses that infect bacteria, utilize the host biosynthetic machinery for replication, and release the progeny bacteriophages with or without the lysis of the infected bacterial cell [229]. Bacteriophages are a potential alternative to antibiotics, as they selectively reduce the abundance of targeted bacterial species and do not affect the normal poultry gut microbiota [230]. However, bacteriophages might not eliminate the target pathogen but reduce their abundance below the critical threshold [231]. Bacteriophage treatment of poultry carcasses is reported to reduce the recovery of Salmonella Enteritidis from the carcass wash by 93% [232], which reduces the risk of colonization of Salmonella in humans upon consumption.

Bacteriophages such as φCJ22 [233], INT401 [234], and φ3626 [235] have been isolated and characterized to evaluate their inhibitory effect on the growth of C. perfringens in poultry. The administration of the polyvalent bacteriophage cocktail INT-401 in chickens effectively improves the weight gain and feed conversion ratio and reduces mortality by 92% during NE challenge [234]. Similarly, *C. perfringens* podovirus phage administration reduces the gross pathology, mortality, and cecal load of *C. perfringens* in infected birds [236]. Moreover, administration of bacteriophages at a concentration of 106–107 plaque-forming units significantly improves weight gain, feed efficiency, and decrease mortality in a NE challenge model. Additionally, bacteriophage treatment reduces the intestinal damage, lesion score, and cecal load of *C. perfringens* in infected birds [233]. These data suggest that bacteriophages can potentially ameliorate the effects of NE in broilers and improve the growth parameters, performance parameters, and mortality associated with NE.

### 11.7. Vaccination

Vaccination is an effective strategy to prevent the prevalence of bacterial and viral diseases in humans and livestock. Vaccines for most clostridial diseases in livestock have been developed based on the toxins involved in the pathogenesis of the particular disease [237]. Nevertheless, until recently, the progress towards developing an effective vaccine for protecting birds from NE has been limited. The study of immunity in broiler chickens to necrotic enteritis has demonstrated that vaccination with virulent strains of *C. perfringens* but not avirulent strains protects birds against necrotic enteritis. Alpha-toxin-deficient strains are also capable of imparting protective immunity, implying that factors other than α-toxin may be responsible for inducing protective immunity [238].

In a study conducted by Thompson et al. [238], birds that recover from NE are immune to subsequent challenge with *C. perfringens*. It was also shown that immunization with attenuated virulent strains completely prevented subsequent infection [238]. In another study using purified recombinant NetB (rNetB) as a vaccine antigen, the birds were significantly protected against a mild oral dose of a virulent strain of *C. perfringens*, but it could not offer protection against a more robust challenge. Subsequently, a vaccine consisting of rNetB, formalin-treated bacterin, and cell free supernatant was used, which provided significant protection to the birds against moderate to severe NE challenge [239]. Immunization of breeder hens with rNetB, toxoid, or a combination of rNetB and toxoid was reported to protect the progeny when challenged at two weeks of age, with the highest level of protection provided by the latter [240]. Vaccinating the chicks against predisposing factors such as coccidiosis, IBD, CIA, and MD will also control the occurrence of NE in birds [241]. Hence, vaccination strategies devised with an in-depth understanding of the virulence factors associated with the development and pathogenesis of NE is essential to prevent the outbreak of NE in poultry.

**Figure 5:**
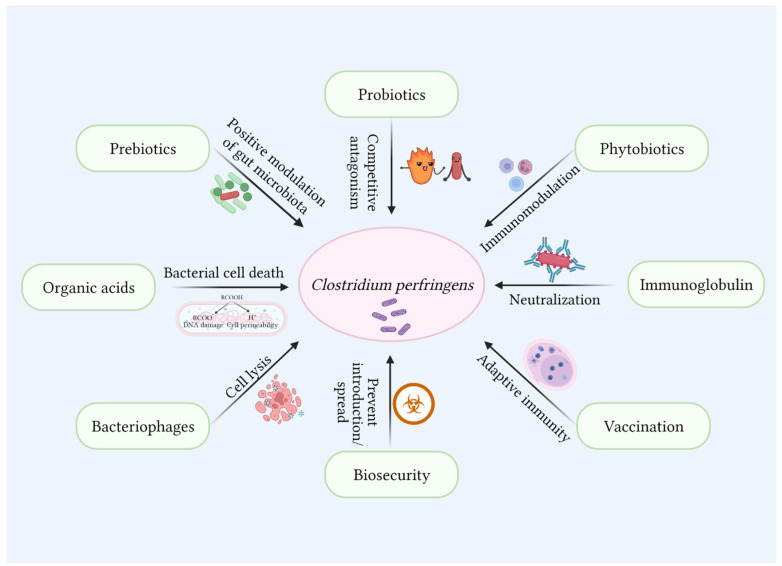
Prevention and control strategies of necrotic enteritis in the post-antibiotic age. Created with Biorender.com (accessed on 24 July 2022).

Together with these alternatives, the choice of ingredients while formulating poultry diet [242], supplementing feed enzymes, and biosecurity measures can also help to reduce the incidence and spread of necrotic enteritis in poultry [243]. Considering the predisposing factors prevalent in an area, alternative strategies consisting of one or a combination of methods can be employed to manage necrotic enteritis in the post-antibiotic era successfully. An abstract of how the various predisposing factors contribute to the outgrowth of *C. perfringens* and predispose the birds to NE is demonstrated in Figure 5.

## 12. Conclusions

The substantial economic burden and public health concerns raised by subclinical and clinical necrotic enteritis demand more research into the pathogenesis of this multifactorial disease. Improved disease models, whole genome sequencing, toxin characterization, and targeted mutation with help to establish a greater understanding of the complex disease process. Thorough knowledge of the virulence factors and pathogenesis is necessary to construct an effective strategy for the prevention of NE. Although several alternatives available in the market reduce the impact of NE on the production performance, further research is essential to establish safety standards for these novel alternatives.

## Figures and Tables

**Table 1:** Classification of *C. perfringens* based on the toxins secreted. In poultry, *C. perfringens* types A, C, and G are responsible for necrotic enteritis. These strains harbor different toxins/enzymes that will be discussed in the following paragraphs.

Toxinotype	Alpha (α)	Beta (β)	Epsilon (ε)	Iota (ι)	CPE	NetB
Type A	+	−	−	−	−	−
Type B	+	+	+	−	−	−
Type C	+	+	−	−	+/−	−
Type D	+	−	+	−	+/−	−
Type E	+	−	−	+	+/−	−
Type F	+	−	−	−	+	−
Type G	+	−	−	−	−	+

**Table 2:** Toxins secreted by various strains of *C. perfringens*, their genetic location and mode of action.

Toxin/Enzyme	Gene Location	Biological Activity	Diseases and Affected Species
Alpha toxin (CPA)	Chromosome	Phospholipase C and sphingomyelinase	Gas gangrene in humans and different animals [18].
Beta toxin (CPB)	Plasmid	Pore-forming toxin	Enterocolitis and Enterotoxaemia in neonatal animals Lamb dysentery struck [19]
Beta2 toxin (CPB2)	Plasmid	Putative pore-forming toxin	Enteric disease in horses [20], pigs [21]
Delta-toxin	Plasmid	Pore-forming toxin	Damages epithelial cells [22]
Enterotoxin (CPE)	Plasmid/ chromosome	Pore-forming toxin	Food poising in humans [23].
Epsilon toxin (ETX)	Plasmid	Pore-forming toxin	Clostridial enterotoxaemia in sheep and goats [24]
NetB (netB)	Plasmid	Pore-forming toxin	Necrotic enteritis [25]
NetE	Plasmid	Putative pore-forming toxin	Acute hemorrhagic diarrhea syndrome in dogs [26]
NetF	Plasmid	Pore-forming toxin	Acute hemorrhagic diarrhea syndrome in dogs [26]
NetG	Plasmid	Putative pore-forming toxin	Acute hemorrhagic diarrhea syndrome in dogs [26]
Theta-toxin/perfringolysin O	Chromosome	Pore-forming toxin	Gas gangrene in humans [27]
Iota toxin (Iap/ibp)	Plasmid	Actin-specific ADP-ribosyl transferase	Enterotoxaemia in rabbits [28]
TepL (TepL)	Plasmid	Ras-specific mono-glucosyltransferase	Necrotic enteritis in chicken [29]
BecA,	Plasmid	Actin-specific ADP-ribosyl transferase	Acute gastroenteritis in humans [30]
BecB	Plasmid	Actin-specific ADP-ribosyl transferase	Acute gastroenteritis in humans [30]
NanH	Chromosome	Sialidase	C. perfringens-mediated tissue infection [31]
NanI	Chromosome	Sialidase	C. perfringens-mediated tissue infection [31]
NanJ	Chromosome	Sialidase	C. perfringens-mediated tissue infection [31]
Kappa-toxin (colA)	Chromosome	Collagenase	
Mu-toxin	Chromosome	Hyaluronidase	
Lambda-toxin	Plasmid	Protease	
Alpha-clostripain	Chromosome	Cysteine protease	

**Table 3:** Biochemical characteristics of *C. perfringens*.

Name of Test	Result
Aerotolerant growth	Negative
Gram staining	Gram positive rods
Motility	Negative
Sheep blood agar (SBA)	Double zone of β-hemolysis
TSC agar	Black color colonies
Hydrogen sulphide	Positive
Nitrate reduction	Reduce nitrate to nitrite
Lecithinase activity	Positive
Gelatin hydrolysis	Positive
Carbohydrate fermentation	Produce acid and gas by the fermentation of glucose, maltose, sucrose, and lactose.
Indole test	Negative
Methyl Red test	Positive
Vogus Proskauer	Negative

**Table 4:** Toll-like receptors on different tissues and cell types of chickens.

Toll-Like Receptor Class	Tissue
TLR1/6/10, TLR2 types 1 and 2, TLR3, TLR4, TLR5, and TLR7 [122]	Heterophils
TLR4, TLR9 [120]	Macrophages
TLR1, TLR3, and TLR6 [123]	Blood, spleen, tonsils, bursa of Fabricius, thymus, liver, kidney, oviduct, lungs, small intestine, and large intestine
TLR2 [123]	Tonsils, blood, spleen, liver, bursa of Fabricius, oviduct, and intestine
TLR4 [123]	Spleen, liver, and tonsils
TLR5 [124]	Tonsils, spleen, liver, lungs, heart, intestine, immune cells, and testis
TLR7 [125]	Spleen, tonsils, bursa
TLR15 [126]	Spleen, liver, bursa, intestine, and tongue
TLR21 [126]	Heterophils

## Data Availability

No new data were created or analyzed in this study. Data sharing is not applicable to this article.
